# Malignant peripheral nerve sheath tumor of the pancreas—A case report

**DOI:** 10.1016/j.ijscr.2019.02.011

**Published:** 2019-02-13

**Authors:** Pradeep Balineni, Rekha Arcot, Kannan Devygounder, Khalilur Rahaman, Bharath Narayansamy, Manoj Prabhu, Shantini Vaitheeswaran

**Affiliations:** Department of General Surgery, Department of Surgical Gastroentrology, Saveetha Medical College Hospital, India

**Keywords:** MPNST of pancreas, Malignant peripheral nerve she, ath tumour, Pancreatic tumour, Schwanomma

## Abstract

Introduction: Nerve sheath tumors are tumors arising from nerve sheaths or which show nerve sheath differentiation. They are divided as benign and malignant. They are associated with Von Recklinghausen syndrome. Characterised by café-au-lait spots, lisch nodules, acoustic neuromas, pheochromocytomas.

Presentation of case: A 62 year old gentleman came with complaints of Abdominal discomfort for 1month. He also had Left sided abdominal pain, Anorexia, Vomiting, No history of dyspepsia, fever, bowel disturbance. Patient is a known case of diabetes mellitus II. On abdominal examination a large mass of 10*8 cms was palpated in the left hypochondrium, left lumbar, and umbilical region. It had restricted mobility, not moving with respiration, firm in consistency. On palpation of neck, the thyroid gland was found to be enlarged with palpable right lobe.

Contrast enhanced computed tomography showed large lobulated heterogenously enhancing mass with internal necrosis and calcifications in the left hypochondrium, in the region of distal body and tail of pancreas. There were no other foci of metastasis in abdomen or chest. Chest X-ray of patient was normal. Ultrasound of neck revealed a suspicious nodule in right lobe of thyroid measuring 1*1 cms with no nodal enlargement. Fnac of thyroid nodule was done under image guidance which was suggestive of papillary carcinoma of thyroid. As image guided biopsy of abdominal tumour could not be done patient was planned for laparotomy. The tumor was resected en bloc - distal pancreatectomy and splenectomy. Post opereative biopsy showed irregular spindle shaped cells with nuclei exhibiting “a wavy, buckled appearance” malignant peripheral nerve sheath tumor. Patient was reviewed after a period of 2 months. Positron emission tomography was done which revealed para aortic nodes and 2 mesenteric nodes. It also revealed a metabolically active nodule in thyroid with cervical nodal metastasis. Patient was taken up for total thyroidectomy with functional neck dissection. Histopathology confirmed papillary carcinoma of thyroid with positive lymph nodes. Patient was advised to undergo a radioactive iodine scan showed 0.3% uptake. Patient was started on chemotherapy for para aortic nodes with a regimen of paclitaxel, adriamycin, ifosumide and mesma.

Discussion: Pancreatic schwannoma arise from sympathetic and Para sympathetic fibers that cross over pancreas. Patients Present with abdominal pain and weight loss commonly. Macroscopically they are well circumscribed, encapsulated lesions, homogenous lesions. Microscopically they show Antoni A & B areas. They demonstrate S100, vimentin, CD56 positivity. Most common differential diagnosis are cystic mucinous / serous neoplasms of pancreas. Malignant transformation is extremely rare with only 8 reported cases till now. Treatment is simple enucleation, but a accurate pre-operative diagnosis is difficult to make hence a oncollogically margin negative resection is done. Adjuvant radiotherapy can be given to reduce the tumor load. Chemotherapy can be given with ifosumide and doxorubicin.

Conclusion: Malignant pancreatic nerve sheath tumours are very rare to be found, but have very poor prognosis, hence in cases of pancreatic tumour its better to keep the diagnosis of nerve sheath tumour also a differential diagnosis. In conclusion pancreatic tumours are to be identified at the earliest and surgery should be done for those cases

## Highlights

• Pancreatic schwannoma arise from sympathetic and Para sympathetic fibers that cross pancreas.
• Macroscopically they are well circumscribed, encapsulated lesions, homogenous lesions.
• Microscopically they show Antoni A & B areas.
• They demonstrate S100, vimentin, CD56 positivity.
• Malignant transformation is extremely rare with only 8 reported cases till now.
• Treatment is simple enucleation, but a accurate pre-operative diagnosis is difficult to make hence a oncollogically margin negative resection is done.

## 1 Case report

A 62 year old gentleman came to the surgical out patient with complaints of abdominal discomfort, occasional left sided abdominal pain for the past 15 days. He had decreased appetite, projectile vomiting, no dyspepsia, no history of fever and bowel habits were normal. On examination, a large mass of 10 × 8 cm was palpated in the left hypochondrium, left lumbar, and umblical region. It was mobile, not moving with respiration and firm in consistency. On palpation of neck, the thyroid gland was found to be enlarged with palpable right lobe. Upper and lower gastrointestinal endoscopy was normal.

**Fig. 1:**
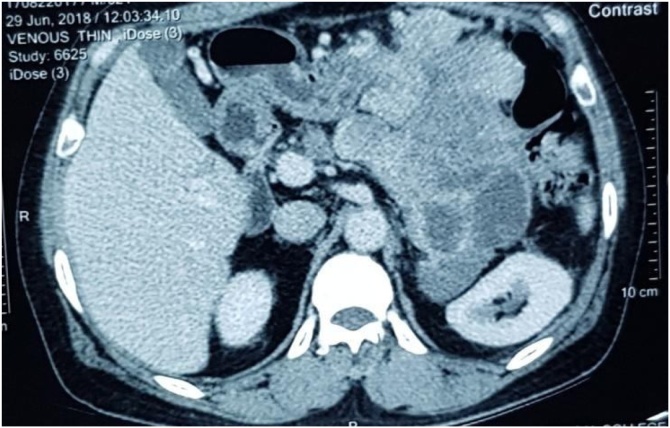
CECT showing the heterogeneously enhancing mass arising from the pancreas.

Contrast enhanced computed tomography (cect) abdomen showed large lobulated, heterogenously enhancing mass with internal necrosis and calcifications in the left hypochondrium in the region of distal body and tail of pancreas (Fig. 1). There were no other foci of metastasis in abdomen or chest. Chest X-ray of patient was normal. Ultrasound of neck revealed a suspicious nodule in right lobe of thyroid measuring 1*1 cm with no nodal enlargement. Pre-operative ultrasound guided biopsy showed features suggested of poorly differentiated malignancy (that was negative for gastro intestinal stromal tumor markers). His CEA and Ca 19-9 were normal. Fine needle aspiration cytology of thyroid nodule was done under image guidance which was suggestive of papillary carcinoma thyroid.

**Fig. 2:**
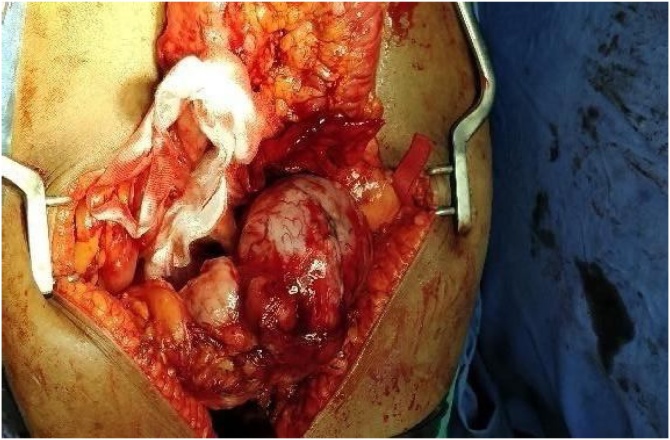
Operative and postoperative image.

As image guided biopsy of abdominal tumour could not be done patient was planned for laparotomy. At laparotomy, patient was found to have a bilobed tumor arising from the lesser sac adherent to the pancreas and abutting the stomach, transverse colon, and left adrenal and splenic hilum (Fig. 2). The tumor was resected en bloc (distal pancreatectomy and splenectomy). The post operative period for the patient was uneventful. Oral feed started on 3^rd^post operative day drain tube was removed on 5^th^post operative day. Patient was discharged on the 12^th^post operative day.

**Fig. 3:**
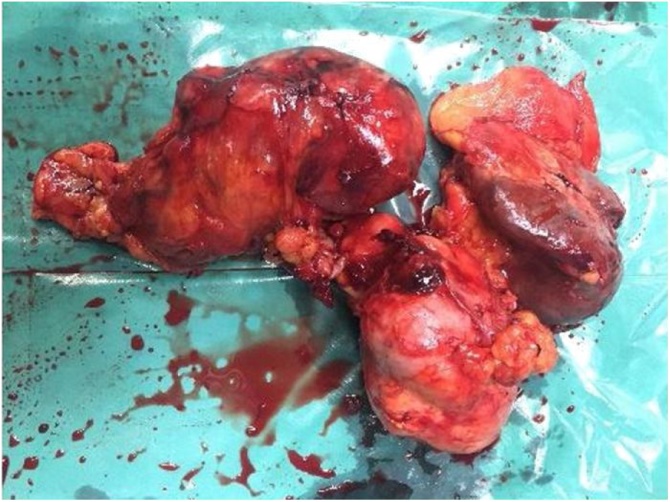
Specimen with tumour and spleen.

**Fig. 4:**
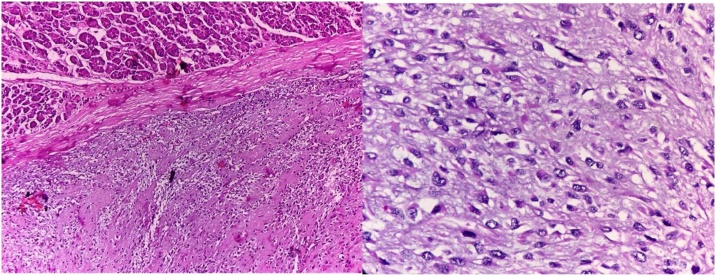
Hematoxylin and eosin staining.

**Fig. 5:**
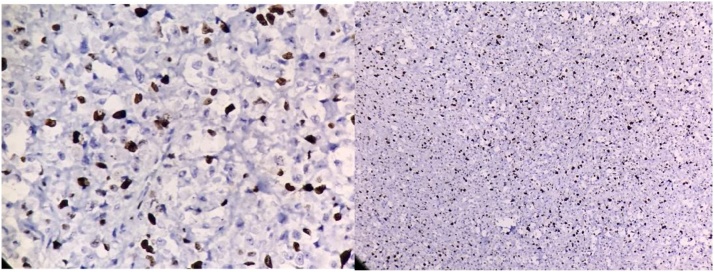
S100 staining.

**Fig. 6:**
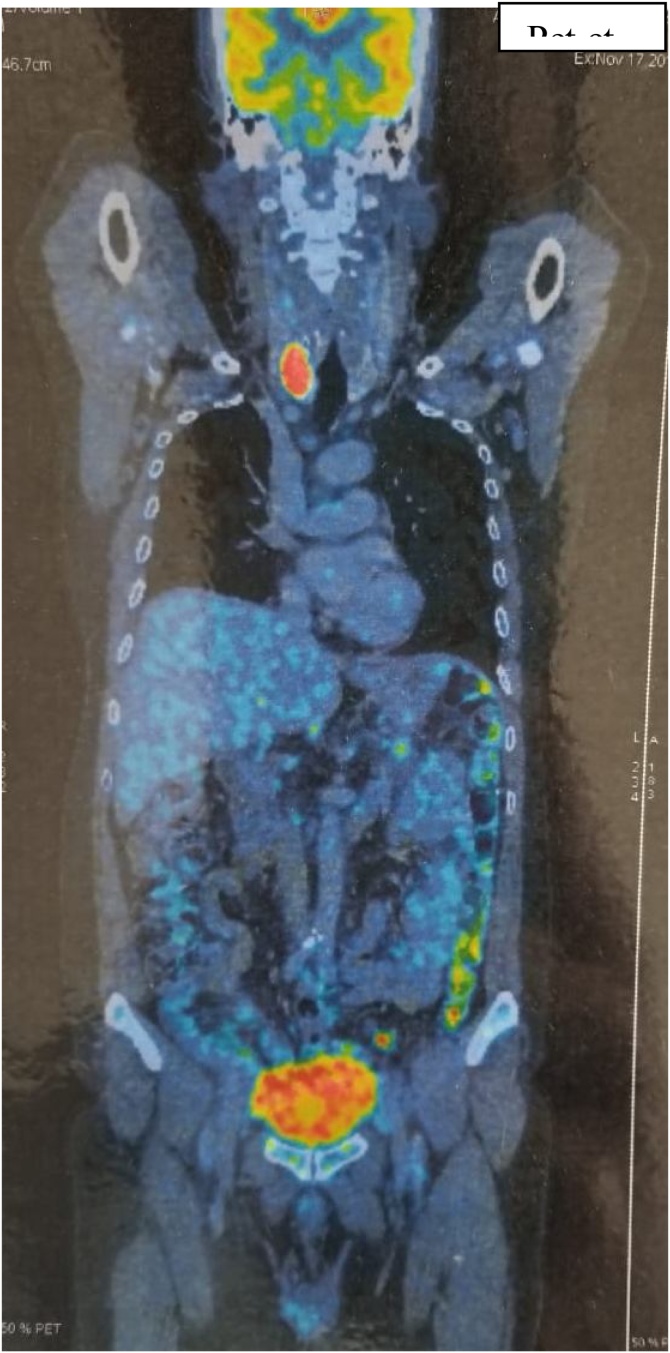
PET scan.

The histopathology showed the presence of very irregularly shaped cells with nuclei that exhibited a “wavy, buckled appearance”. Multiple sections studied showed pancreatic tissue with adjacent fairly circumscribed neoplasm composed of spindle cells arranged in sheets and fascicles. Pancreatic margin appeared free from tumour. There are areas showing alveolar and glandular patterns.There are areas showing hyalinization, necrosis and fibrosis. Tissue organization shows great variability, with hypocellular myxoid areas and areas of major cellularity diagnostic of Malignant peripheral nerve sheath tumour (MPNST). The histologic changes of MPNST include spindle cells with comma-shaped nuclei, tactoid bodies, nuclear palisading, hyaline bands, and schwannoma-like and curlicue foci (Fig. 3).The tumour showed focal S100 positivity,was strongly positive for ki67, was positive for vimentin (Figs. 4 and 5) and negative for cytokeratin,synaptophysin and all GIST markers(CD 117,DOG 1,PDGF) We re-examined the patient thoroughly and there were no cutaneous markers for neurofibromatosis and examination of the eye was normal. Patient was reviewed after a period of 2 months. Positron emission tomography (Fig. 6) was done which revealed para aortic nodes and 2 mesentric nodes. It also revealed a metabolically active nodule in thyroid with cervical nodal metastasis. Patient was taken up for total thyroidectomy with functional neck dissection. Histopathology confirmed papillary carcinoma of thyroid with positive lymph nodes. Patient was advised to undergo a radioactive iodine scan which showed 0.3% uptake. Hence patient was started on chemotherapy for para aortic nodes with a regimen of paclitaxel, adriamycin, ifosumide and mesma. Patient has completed 3 cycles till date and is on regular follow up.

## 2 Discussion

Neural tumours are schwannomas and neurofibromas [1] They are associated with von Recklinghausen syndrome and often have the spectrum of café-au-lait spots, acoustic neuromas, phaeochromocytoma, Lisch nodules (pigmented hamartomas of the iris) and skeletal malformations. Malignant peripheral nerve sheath tumors (MPNSTs) are rare and highly aggressive neoplasms, representing only 5% of soft tissue sarcomas. MPNSTs may appear *de novo* or develop from the malignant transformation of a benign neural neoplasm, generally a plexiform neurofibroma. Approximately half of MPNST cases occur in association with neurofibromatosis type 1 (NF1).Solitary (unassociated with NF1) and localized (or discrete; multiple in NF1) neurofibromas do not have malignant transformation potential. MPNST originating from the bone, soft tissues, heart, mouth, esophagus, bronchus, retroperitoneum, uterine cervix, orbit, parotid gland, as well as the spinal cord, acoustic nerve, cerebellum, and sympathetic chain, have been reported.

Schwanommas and neurofibroma have been reported to occur on the pancreas infrequently [[2], [3], [4]]. Malignant transformation occurs in less than 1% of tumors and malignant peripheral nerve sheath tumors (MPNST) are uncommon on the pancreas and the lesser sac.*TP53* mutations have been found in a subgroup of MPNSTs, indicating that a p53-mediated pathway is involved in their development [5,6].

MPNST is typically associated with a poor outcome compared with those of other soft tissue sarcomas. The recurrence rate is as high as 40%, and the most common metastatic sites are the lungs and the bone. The 5-year survival rate ranges from 30 to 50%. The negative prognostic factors for MPNST are similar to those for other soft tissue sarcomas and include the tumor site (head, neck, and trunk vs. the extremities), a large tumor size, high-grade, and positive surgical margins. Some reports suggested that NF1 patients with MPNST experience poorer survival than do sporadic MPNST patients although this has not been a consistent finding [7]. Complete surgical resection is the mainstay of MPNST treatment. Incomplete surgical resection increases the risk of MPNST-specific death nearly six-fold. Adjuvant radiotherapy may improve local tumor control; however, any evidence that survival is prolonged by radiotherapy is limited. If the preoperative diagnosis had been correct (identification of MPNST with a biopsy based on immunohistochemical markers [8]), neoadjuvant radiotherapy may have reduced the tumor volume, rendering complete excision possible. Role of chemotherapy for MPNST was proved more in pediatric patients than in adults. The overall response rate to primary chemotherapy was reported as 45% in a group of pediatric MPNST patients. First-line chemotherapy typically consists of a combination of ifosfamide and doxorubicin.There have been fewer than 200 cases of PNST reported since it was first described in 1932, with approximately 40 reported malignancies as of 2014 [9].

Written informed consent was obtained from the patient for publication of this case report and accompanying images. A copy of the written consent is available for review by the Editor-in-Chief of this journal on request.

## Conflicts of interest

No conflict of interests.

## Funding

No sources, self funding.

## Ethical approval

The case report is exempted from ethical committee.

## Consent

Consent was obtained from patient for use of clinical information for academic and Educational purposes. A copy of the written consent is available for review by the Editor-in-Chief of this journal on request.

## Author contribution

Study conception and design: Dr. Rekha Arcot ; Dr.Khalilur rahman ; Dr. Bharath Narayansamy.

Acquisition of data: Dr.Pradeep Balineni ; Dr. Manoj Prabhu K.R ;Dr. Shantini Vaitheeswaran.

Analysis and interpretation of data: Dr. Kannan Devydounder.

Drafting of manuscript: Dr. Rekha Arcot ; Dr.Pradeep Balineni ; Dr. Bharath Narayansamy.

Critical revision: Dr.Pradeep Balineni ; Dr. Rekha Arcot ; Dr. Manoj Prabhu K.R.

## Registration of research studies

Not applicable.

## Guarantor

Dr. Pradeep Balineni.

Dr. Rekha Arcot.

## Provenance and peer review

Not commissioned, externally peer-reviewed.
